# Robust Inverse Reconstruction of Time-Varying Transmission Rates Across Model Structures and Incidence Forms

**DOI:** 10.1007/s11538-026-01597-4

**Published:** 2026-01-29

**Authors:** Xiunan Wang, Hao Wang

**Affiliations:** 1 Department of Mathematics, University of Tennessee at Chattanooga, Chattanooga, TN 37403 USA; 2 Department of Mathematical and Statistical Sciences, University of Alberta, Edmonton, AB T6G2G1 Canada

## Abstract

Accurate, decision-ready estimates of time-varying transmission rates are critical, yet thought to be sensitive to model specification. We test this sensitivity by applying a continuous inverse method to weekly influenza and measles data, comparing reconstructions across eight common compartmental structures (SIS/SIR/SEIS/SEIR and vaccinated variants) and across five incidence forms (mass action vs. saturated). Timing and ordering of peaks and troughs in the transmission rates are highly consistent across influenza models, with amplitude shifts matching mechanistic expectations (attenuation with vaccination; smoothing with latent periods). For measles, we show that the transmission rates under saturated incidence preserve the rise-and-fall ordering observed under mass action and provide a sufficient condition ensuring matched monotonicity. These results indicate inverse transmission rate reconstructions are robust to typical structural and incidence choices, supporting their routine use for interpreting transmission dynamics, short-term forecasting, and intervention assessment.

## Introduction

Estimating the transmission rate of an infectious disease is essential for epidemic forecasting and guiding public health interventions, as it reflects how rapidly a pathogen spreads and underlies the calculation of the effective reproduction number. Our perception of a disease’s contagiousness or severity often relies on reported case or mortality time series; however, these data do not always accurately represent the true transmissibility. For instance, when the number of reported cases is small, the disease may have already spread extensively with a high transmission rate, especially if many individuals are still in the incubation or asymptomatic phase and thus remain undetected. In contrast, when case numbers appear large, the actual transmission rate might have declined due to increased public awareness and preventive behavior (Wang and Wang 2024). Therefore, it is crucial to develop mathematical approaches capable of inferring transmission rates from observed epidemiological data, providing valuable insights into the dynamics of disease spread and the effectiveness of control measures.

A number of studies have proposed approaches for estimating time-varying transmission rates within the framework of differential equation models (see, e.g., Pollicott et al. (2012); Hadeler (2011); Jagan et al. (2020); Mubayi et al. (2021)). Pollicott et al. (2012) first introduced a continuous inverse method that determines the transmission rate by solving a Bernoulli differential equation derived from the model system. This approach was subsequently extended by Hadeler (2011) and Mubayi et al. (2021) to accommodate both prevalence and incidence data. Building on this framework, Kong et al. (2015) developed inverse method algorithms tailored to a childhood measles model, applying them to both pre-vaccination and post-vaccination datasets. Jagan et al. (2020) proposed a rapid approach to estimate time-varying transmission rates from incidence time series, introducing a “peak-to-peak” iterative technique that effectively reduces sensitivity to the assumed initial number of susceptibles. More recently, we developed a discrete inverse method which is particularly powerful when the incidence function does not explicitly depend on the transmission rate (as is the case for certain diseases with an incubation period) and demonstrated its application to diseases with different cycles (Wang and Wang 2024). The discrete inverse method was further employed to integrate differential equation models with machine learning, enhancing forecasting accuracy for daily COVID-19 cases in the United States (Wang and Wang 2024; Wang et al. 2022a, b; Chakraborty et al. 2024).

Although inverse methods provide a powerful mathematical framework for estimating transmission rates from observed disease incidence data (Wang and Wang 2024; Pollicott et al. 2012), the extent to which these estimates depend on the choice of epidemiological model remains unclear. Different compartmental structures (e.g., SIS, SIR, SEIS models) incorporate distinct assumptions about disease progression, potentially influencing the inferred transmission rates. Likewise, variations in incidence formulations (e.g., mass action, Holling type I, or Holling type II) may also affect the resulting estimates. Understanding how model structure and incidence function shape transmission rate estimation is therefore essential for developing reliable and generalizable forecasting frameworks. To our knowledge, a systematic comparison of inverse-method estimates across different model structures and incidence functions has not yet been undertaken.

In this study, we fill this gap by comparing the transmission rates estimated using the continuous inverse method across two groups of models. For the first group, we examine flu models with different compartmental structures (SIS, SIR, SEIS, SEIR, SISV, SIRV, SEISV, and SEIRV), while for the second group we analyze measles models with the same structure but with different incidence functions, including mass action, Holling type II, Beddington–DeAngelis, and other nonlinear forms. By comparing the resulting transmission rate curves, we assess how model selection influences the inferred temporal patterns. Our results show that the monotonicity and timing of peaks and troughs are remarkably consistent across models, indicating that the inverse method robustly captures the temporal dynamics of transmission independent of specific structural or functional choices. This robustness highlights the method’s potential as a reliable framework for studying transmission variability in diverse epidemiological contexts.

The rest of this paper is structured as follows. In Section 2, we compare transmission rate estimates across different flu compartmental models. In Section 3, we compare transmission rate estimates across measles models with different incidence functions. In both these two sections, we first introduce the disease transmission mechanisms and formulate the models, then we show the algorithms for deriving the transmission rates inversely from epidemic data, and eventually present the simulation results comparing the transmission rate estimates across these models. In Appendix 1, we identify the condition and potential mechanisms underlying the observed phenomena in the measles model with two different incidence functions. Section 4 provides a discussion of the implications and concludes with directions for future work.

## Comparing Transmission Rate Estimates Across Model Structures

### Flu Models with Different Compartmental Structures

Since influenza dynamics can be represented by a variety of compartmental models that differ in their assumptions about immunity, latency, and vaccination, it is important to evaluate how these structural choices affect the transmission rate estimated via the inverse method. Here, we develop flu models with eight different structures (SIS, SIR, SEIS, SEIR, SISV, SIRV, SEISV, SEIRV) to assess the robustness of inference and to clarify the implications of modeling assumptions for influenza transmission dynamics. The variables *S*(*t*), *E*(*t*), *I*(*t*), *R*(*t*), *V*(*t*) represent susceptible, exposed, infectious, recovered and vaccinated population at time *t*, respectively. The SIS model assumes that individuals lose immunity and return to the susceptible pool after recovery, which may approximate flu dynamics given waning immunity. Even within a single flu season, it is possible for a person who has recovered from an infection with one flu strain to be infected with a different strain. Thus, it is reasonable to consider SIS type models without the recovered compartment. The SIR model incorporates permanent recovery-induced immunity, a reasonable approximation for many infectious diseases but less realistic for influenza. The SEIS and SEIR models extend these frameworks by including an exposed (E) compartment, representing the latent period before individuals become infectious, which is critical for influenza given its incubation phase. The SISV, SIRV, SEISV, and SEIRV models include a vaccinated (V) compartment and assume that the vaccine-induced immunity lasts throughout the flu season, with vaccinated individuals having a reduced risk of infection compared to those in the susceptible compartment. The formulations of the flu models are given as follows:

- SIS model:
  1 $$\begin{aligned} \begin{aligned} S'(t)&= -\frac{\beta (t) S(t)I(t)}{N(t)}+\eta I(t),\\ I'(t)&= \frac{\beta (t) S(t)I(t)}{N(t)}-\eta I(t)-\mu (t)I(t). \end{aligned} \end{aligned}$$
- SIR model:
  2 $$\begin{aligned} \begin{aligned} S'(t)&= -\frac{\beta (t) S(t)I(t)}{N(t)},\\ I'(t)&= \frac{\beta (t) S(t)I(t)}{N(t)}-\eta I(t)-\mu (t)I(t), \\ R'(t)&= \eta I(t). \end{aligned} \end{aligned}$$
- SEIS model:
  3 $$\begin{aligned} \begin{aligned} S'(t)&= -\frac{\beta (t) S(t)I(t)}{N(t)}+\eta I(t),\\ E'(t)&= \frac{\beta (t) S(t)I(t)}{N(t)}-\xi E(t),\\ I'(t)&= \xi E-\eta I(t)-\mu I(t). \\ \end{aligned} \end{aligned}$$
- SEIR model:
  4 $$\begin{aligned} \begin{aligned} S'(t)&= -\frac{\beta (t) S(t)I(t)}{N(t)},\\ E'(t)&= \frac{\beta (t) S(t)I(t)}{N(t)}-\xi E(t),\\ I'(t)&= \xi E(t)-\eta I(t)-\mu (t) I(t),\\ R'(t)&= \eta I(t). \end{aligned} \end{aligned}$$
- SISV model:
  5 $$\begin{aligned} \begin{aligned} S'(t)&= -\frac{\beta (t) S(t)I(t)}{N(t)}-\nu (t) S(t)+\eta I(t),\\ I'(t)&= \frac{\beta (t) (S(t)+\epsilon V(t))I(t)}{N(t)}-\eta I(t)-\mu (t)I(t), \\ V'(t)&= \nu (t) S(t)-\frac{\epsilon \beta (t) V(t)I(t)}{N(t)}. \end{aligned} \end{aligned}$$
- SIRV model:
  6 $$\begin{aligned} \begin{aligned} S'(t)&= -\frac{\beta (t) S(t)I(t)}{N(t)}-\nu (t) S(t),\\ I'(t)&= \frac{\beta (t) (S(t)+\epsilon V(t))I(t)}{N(t)}-\eta I(t)-\mu (t)I(t), \\ R'(t)&= \eta I(t),\\ V'(t)&= \nu (t) S(t)-\frac{\epsilon \beta (t) V(t)I(t)}{N(t)}. \end{aligned} \end{aligned}$$
- SEISV model:
  7 $$\begin{aligned} \begin{aligned} S'(t)&= -\frac{\beta (t) S(t)I(t)}{N(t)}-\nu (t) S(t)+\eta I(t),\\ E'(t)&= \frac{\beta (t) (S(t)+\epsilon V(t))I(t)}{N(t)}-\xi E(t),\\ I'(t)&= \xi E-\eta I(t)-\mu (t) I(t), \\ V'(t)&= \nu (t) S(t)-\frac{\epsilon \beta (t) V(t)I(t)}{N(t)}. \end{aligned} \end{aligned}$$
- SEIRV model:
  8 $$\begin{aligned} \begin{aligned} S'(t)&= -\frac{\beta (t) S(t)I(t)}{N(t)}-\nu (t) S(t),\\ E'(t)&= \frac{\beta (t) (S(t)+\epsilon V(t))I(t)}{N(t)}-\xi E(t),\\ I'(t)&= \xi E(t)-\eta I(t)-\mu (t) I(t),\\ R'(t)&= \eta I(t),\\ V'(t)&= \nu (t) S(t)-\frac{\epsilon \beta (t) V(t)I(t)}{N(t)}. \end{aligned} \end{aligned}$$

The transmission rate $\beta (t)$, the vaccination rate $\nu (t)$ and the disease-induced death rate $\mu (t)$ are all time-varying parameters. The total population *N*(*t*) can also vary with time and we take a different value for *N*(*t*) for a different flu season. The recovery rate $\eta$ is assumed to be constant. Since vaccines against influenza provide only partial protection (Centers for Disease Control 2025a), both susceptible and vaccinated individuals could be infected by contacting an infectious individual. The relative risk of infection for vaccinated individuals compared to susceptible ones is $\epsilon$. The parameters and their interpretations in these flu models are given in Table 1.

**Table 1:** Interpretations of parameters for models (1)-(8)

Parameter	Interpretation
$\beta (t)$	transmission rate
$\eta$	recovery rate
$\mu (t)$	disease-induced death rate
$1/\xi$	incubation period
$\nu (t)$	vaccination rate
$\epsilon$	relative risk of infection for vaccinated individuals

### Extracting Flu Transmission Rates Using Inverse Method

In order to use the continuous inverse method to estimate the transmission rates, we need to obtain a spline of the time series of the weekly new infections. We denote this splined function as *y*(*t*). Similarly, we can also obtain the splined function of disease-induced deaths each week, denoted as *d*(*t*) and that of the vaccinated population each week, denoted as *v*(*t*). Then we can replace $\mu (t)I(t)$ by *d*(*t*) and replace $\nu (t) S(t)$ by *v*(*t*) in the flu models. If the model does not include the exposed compartment *E*(*t*), then the disease incidence function can be represented by *y*(*t*). If the model includes an exposed compartment *E*(*t*), we assume that only individuals who develop symptoms are recorded in the weekly count of new infections. In this case, the disease incidence cannot be directly represented by *y*(*t*). Instead, we have $\xi E(t)=y(t)$. The derivations of the transmission rate $\beta (t)$ for the flu models with different compartmental structures are given as follows:

- For the SIS model (1), $y(t)=\frac{\beta (t)S(t)I(t)}{N(t)}$. We can solve the following system to obtain *S*(*t*) and *I*(*t*):
  $$\begin{aligned} { \begin{aligned} S'(t)&= -y(t)+\eta I(t),\\ I'(t)&= y(t)-\eta I(t)-d(t). \end{aligned} } \end{aligned}$$
  Then we obtain
  $$\begin{aligned} \beta (t)=\frac{N(t)y(t)}{S(t)I(t)}. \end{aligned}$$
- For the SIR model (2), $y(t)=\frac{\beta (t)S(t)I(t)}{N(t)}$. We can solve the following system to obtain *S*(*t*) and *I*(*t*):
  $$\begin{aligned} { \begin{aligned} S'(t)&= -y(t),\\ I'(t)&= y(t)-\eta I(t)-d(t). \end{aligned} } \end{aligned}$$
  Then we obtain
  $$\begin{aligned} \beta (t)=\frac{N(t)y(t)}{S(t)I(t)}. \end{aligned}$$
- For the SEIS model (3), $y(t)=\xi E(t)$, then $E(t)=\frac{y(t)}{\xi }$. We can solve for *I*(*t*) from the equation
  $$\begin{aligned} I'(t) = y(t)-\eta I(t)-d(t). \end{aligned}$$
  From the equation $E'(t)=\frac{\beta (t)S(t)I(t)}{N(t)}-\xi E(t)$, we have
  $$\begin{aligned} \frac{\beta (t)S(t)I(t)}{N(t)}=E'(t)+\xi E(t)=\frac{y'(t)}{\xi }+y(t). \end{aligned}$$
  Then we can solve for *S*(*t*) from the equation $S'(t) = -\frac{y'(t)}{\xi }-y(t)+\eta I(t)$. It follows that
  $$\begin{aligned} \beta (t)=\frac{N(t)y'(t)}{\xi S(t)I(t)}+\frac{N(t)y(t)}{S(t)I(t)}. \end{aligned}$$
- For the SEIR model (4), $y(t)=\xi E(t)$, then $E(t)=\frac{y(t)}{\xi }$. We can solve for *I*(*t*) from the equation
  $$\begin{aligned} I'(t) = y(t)-\eta I(t)-d(t). \end{aligned}$$
  From the equation $E'(t)=\frac{\beta (t)S(t)I(t)}{N(t)}-\xi E(t)$, we have
  $$\begin{aligned} \frac{\beta (t)S(t)I(t)}{N(t)}=E'(t)+\xi E(t)=\frac{y'(t)}{\xi }+y(t). \end{aligned}$$
  Then we can solve for *S*(*t*) from the equation $S'(t) = -\frac{y'(t)}{\xi }-y(t)$. It follows that
  $$\begin{aligned} \beta (t)=\frac{N(t)y'(t)}{\xi S(t)I(t)}+\frac{N(t)y(t)}{S(t)I(t)}. \end{aligned}$$
- For the SISV model (5), $y(t)=\frac{\beta (t)(S(t)+\epsilon V(t))I(t)}{N(t)}$. We can solve for *S*(*t*), *I*(*t*) and *V*(*t*) from the following system:
  $$\begin{aligned} \begin{aligned} S'(t)&= -\frac{S(t)y(t)}{S(t)+\epsilon V(t)}-v(t)+\eta I(t), \\ I'(t)&= y(t)-\eta I(t)-d(t),\\ V'(t)&=v(t)-\frac{\epsilon V(t)y(t)}{S(t)+\epsilon V(t)}. \end{aligned} \end{aligned}$$
  Then we obtain
  $$\begin{aligned} \beta (t)=\frac{N(t)y(t)}{(S(t)+\epsilon V(t))I(t)}. \end{aligned}$$
- For the SIRV model (6), $y(t)=\frac{\beta (t)(S(t)+\epsilon V(t))I(t)}{N(t)}$. We can solve for *S*(*t*), *I*(*t*) and *V*(*t*) from the following system:
  $$\begin{aligned} \begin{aligned} S'(t)&= -\frac{S(t)y(t)}{S(t)+\epsilon V(t)}-v(t), \\ I'(t)&= y(t)-\eta I(t)-d(t),\\ V'(t)&=v(t)-\frac{\epsilon V(t)y(t)}{S(t)+\epsilon V(t)}. \end{aligned} \end{aligned}$$
  Then we obtain
  $$\begin{aligned} \beta (t)=\frac{N(t)y(t)}{(S(t)+\epsilon V(t))I(t)}. \end{aligned}$$
- For the SEISV model (7), $y(t)=\xi E(t)$, then $E(t)=\frac{y(t)}{\xi }$. We can solve for *I*(*t*) from the equation
  $$\begin{aligned} I'(t) = y(t)-\eta I(t)-d(t). \end{aligned}$$
  From the equation $E'(t)=\frac{\beta (t)(S(t)+\epsilon V(t))I(t)}{N(t)}-\xi E(t)$, we have
  $$\begin{aligned} \frac{\beta (t)(S(t)+\epsilon V(t))I(t)}{N(t)}=E'(t)+\xi E(t)=\frac{y'(t)}{\xi }+y(t). \end{aligned}$$
  Then we can solve for *S*(*t*) and *V*(*t*) from the following system:
  $$\begin{aligned} \begin{aligned} S'(t)&= -\left( \frac{y'(t)}{\xi }+y(t)\right) \frac{S(t)}{S(t)+\epsilon V(t)}-v(t)+\eta I(t),\\ V'(t)&= v(t)-\left( \frac{y'(t)}{\xi }+y(t)\right) \frac{\epsilon V(t)}{S(t)+\epsilon V(t)}. \end{aligned} \end{aligned}$$
  It follows that
  $$\begin{aligned} \beta (t)=\frac{N(t)y'(t)}{\xi (S(t)+\epsilon V(t))I(t)}+\frac{N(t)y(t)}{(S(t)+\epsilon V(t))I(t)}. \end{aligned}$$
- For the SEIRV model (8), $y(t)=\xi E(t)$, then $E(t)=\frac{y(t)}{\xi }$. We can solve for *I*(*t*) from the equation
  $$\begin{aligned} I'(t) = y(t)-\eta I(t)-d(t). \end{aligned}$$
  From the equation $E'(t)=\frac{\beta (t)(S(t)+\epsilon V(t))I(t)}{N(t)}-\xi E(t)$, we have
  $$\begin{aligned} \frac{\beta (t)(S(t)+\epsilon V(t))I(t)}{N(t)}=E'(t)+\xi E(t)=\frac{y'(t)}{\xi }+y(t). \end{aligned}$$
  Then we can solve for *S*(*t*) and *V*(*t*) from the following system:
  $$\begin{aligned} \begin{aligned} S'(t)&= -\left( \frac{y'(t)}{\xi }+y(t)\right) \frac{S(t)}{S(t)+\epsilon V(t)}-v(t),\\ V'(t)&= v(t)-\left( \frac{y'(t)}{\xi }+y(t)\right) \frac{\epsilon V(t)}{S(t)+\epsilon V(t)}. \end{aligned} \end{aligned}$$
  It follows that
  $$\begin{aligned} \beta (t)=\frac{N(t)y'(t)}{\xi (S(t)+\epsilon V(t))I(t)}+\frac{N(t)y(t)}{(S(t)+\epsilon V(t))I(t)}. \end{aligned}$$

#### Remark 1

We do not need to derive the analytic solutions in order to extract transmission rates using the continuous inverse method. Instead, we can directly follow the procedures described above to solve the model equations numerically using MATLAB ode45 to obtain the time series of the variables.

### Comparing Flu Transmission Rates

**Fig. 1:**
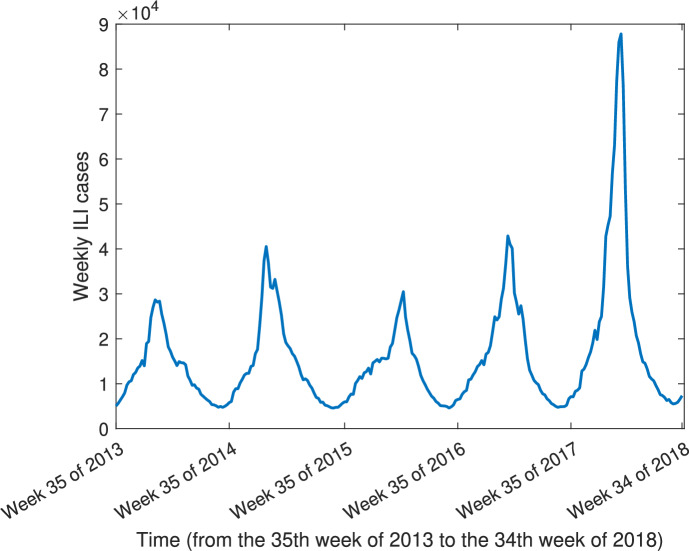
Weekly reported ILI cases in the US from the 35th week of 2013 to the 34th week of 2018. Data source: Centers for Disease Control (2025b)

The weekly reported influenza-like illness (ILI) incidence shown in Figure 1 is used as the primary observational data to estimate flu transmission rates.

The parameter values are chosen to be consistent with those used in our previous study (Wang and Wang 2024), where they were justified based on CDC influenza data and epidemiological evidence. Specifically, we adopt an average infectious period of 7 days ($\eta = 1$ per week) and a vaccine effectiveness of $\epsilon = 0.5$. We do not need to estimate the vaccination rate $\nu (t)$ and mortality rate $\mu (t)$ since we directly use the splined functions *v*(*t*) and *d*(*t*) based on the weekly vaccination data and mortality data from CDC (Centers for Disease Control 2025a), as described in Wang and Wang (2024). The total population size *N*(*t*) is adjusted by flu season following annual U.S. census estimates (United States Census Bureau 2025) as shown in Table 2. In addition, to account for the latent stage of infection introduced in the SEIS, SEIR, SEISV, SEIRV models, we assume an average incubation period of 2 days, that is, $\xi = 3.5$ per week (Centers for Disease Control 2025a).

**Table 2:** Total population of the United States for each flu season year

Flu seasons	2013-2014	2014-2015	2015-2016	2016-2017	2017-2018
Population	316129000	319113000	321442000	323100000	325719000

**Fig. 2:**
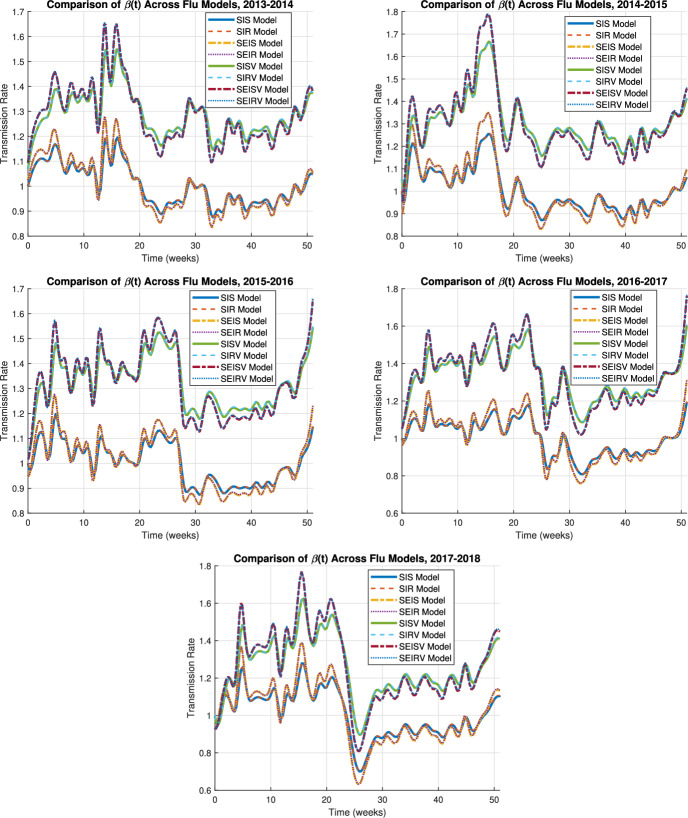
Transmission rates $\beta (t)$ for influenza in five different flu seasons across models (1) to (8)

Figure 2 compares the extracted transmission rates $\beta (t)$ for influenza in five different flu seasons across models (1) to (8).

Despite differences in model structures, all models exhibit similar temporal oscillations in $\beta (t)$. Although the absolute magnitudes of $\beta (t)$ vary depending on model complexity, the overall qualitative trends, including monotonic increases and decreases, remain robust and consistent among models. The timing of peaks and troughs is well aligned across model types, indicating that each model structure captures the same seasonal pattern of influenza transmission.

Introducing a recovered compartment does not alter the temporal trend of the estimated transmission rates. Each model pair (with and without a recovered class) shows nearly identical trajectories: SIS aligns with SIR, SEIS aligns with SEIR, SISV aligns with SIRV, and SEISV aligns with SEIRV. This consistency suggests that inclusion of recovery does not substantially influence the inferred temporal dynamics of $\beta (t)$.

In contrast, models incorporating an exposed (latent) compartment yield systematically higher transmission rate estimates than their corresponding models without it. Specifically, $\beta (t)$ values for SEIS exceed those of SIS, SEIR exceed SIR, SEISV exceed SISV, and SEIRV exceed SIRV. This difference likely arises because the exposed compartment introduces a delay between infection and infectiousness, requiring higher effective transmission rates to reproduce observed incidence data.

Models that include vaccination (SISV, SIRV, SEISV, SEIRV) consistently produce higher estimates of $\beta (t)$ compared to their non-vaccination counterparts (SIS, SIR, SEIS, SEIR). This occurs because vaccination reduces the susceptible population and lowers the infection risk of vaccinated individuals, effectively decreasing the denominator in the transmission expression from *S*(*t*) to $S(t)+\epsilon V(t)$ and, as a result, inflating the inferred $\beta (t)$. Note that this does not mean vaccination increases actual transmission rates. Rather, to produce the same observed incidence of new infections, a population with vaccination must have higher inferred transmission rates, because vaccination reduces the number of susceptible individuals.

#### Remark 2

Since the data are recorded on a weekly basis, the time points are inherently discrete with a resolution of one week. Therefore, when investigating the timing of maximum or minimum values of the transmission rates, it is sufficient to record the values up to one decimal place, corresponding to approximately 0.7 days. Reporting more digits would not increase accuracy because the underlying measurements do not provide finer temporal resolution, and could give a misleading sense of precision.

## Comparing transmission rate estimates across incidence functions

### Measles Models with Different Incidence Functions

We will compare the transmission rates estimated from measles models that employ different incidence functions but share the same model structure as the SEIRA model described in Kong et al. (2015):

9 $$\begin{aligned} { \begin{aligned} \frac{\textrm{d}S(t)}{\textrm{d}t}&= \lambda A(t)-\omega (t)-g S(t),\\ \frac{\textrm{d}E(t)}{\textrm{d}t}&= \omega (t)-\gamma E(t)-gE(t),\\ \frac{\textrm{d}I(t)}{\textrm{d}t}&= \gamma E(t)-\delta I(t)-g I(t), \\ \frac{\textrm{d}R(t)}{\textrm{d}t}&= \delta I(t)-g R(t),\\ \frac{\textrm{d}A(t)}{\textrm{d}t}&= g(S(t)+E(t)+I(t)+R(t))-\lambda A(t),\\ \end{aligned} } \end{aligned}$$

where the variables *S*(*t*), *E*(*t*), *I*(*t*), and *R*(*t*) represent the susceptible, exposed, infectious, and recovered compartments of non-adult individuals, respectively, and *A*(*t*) represents the adult population. Considering that measles is mainly a childhood disease, we assume that adults will not get infected. The disease incidence function is denoted by $\omega (t)$ which can be approximated by a spline curve of the reported data of new infections. The maturation rate from the non-adult stage to the adult stage is denoted by *g*. The incubation period has a duration of $1/\gamma$. The recovery rate is denoted by $\delta$. Both the birth rate and the natural death rate are assumed to be $\lambda$. We consider the following different incidence functions:

- The mass action incidence function from Kong et al. (2015):
  10 $$\begin{aligned} \omega (t)=\beta (t)S(t)I(t). \end{aligned}$$
- Holling type II incidence function from Capasso and Serio (1978):
  11 $$\begin{aligned} \omega (t)=\frac{\beta (t)S(t)I(t)}{1+\alpha I(t)}. \end{aligned}$$
- A specific nonlinear incidence function from Xiao and Ruan (2007):
  12 $$\begin{aligned} \omega (t)=\frac{\beta (t)S(t)I(t)}{1+\alpha I(t)^2}. \end{aligned}$$
- A general nonlinear incidence function from Capasso and Serio (1978); Hethcote and van den Driessche (1991):
  13 $$\begin{aligned} \omega (t)=\frac{\beta (t)S(t)I(t)^p}{1+\alpha I(t)^q}. \end{aligned}$$
- Beddington-DeAngelis incidence function from Liu and Wei (2022):
  14 $$\begin{aligned} \omega (t)=\frac{\beta (t)S(t)I(t)}{1+\alpha _1S(t)+\alpha _2I(t)}. \end{aligned}$$

The mass action incidence function (10) represents the classical assumption that new infections occur through random contacts between susceptible and infectious individuals. The formulation assumes that each susceptible has an equal probability of contacting any infectious individual and that the number of new infections is proportional to the product of the susceptible and infectious populations. This bilinear form captures the simplest form of transmission dynamics, without accounting for saturation effects, behavioral changes, or heterogeneous mixing patterns, and serves as a baseline for comparison with more complex nonlinear incidence functions.

The Holling type II incidence function (11) represents a saturation effect in disease transmission as the number of infectious individuals increases. Here, $\alpha$ measures the strength of the saturation. Biologically, this form reflects the idea that the rate of new infections does not grow indefinitely with *I*(*t*) because susceptibles have a limited capacity to make effective contacts, or because behavioral changes, medical interventions, or crowding effects reduce the per-capita transmission at high infection levels. Compared to the mass action incidence, the Holling type II function captures more realistic transmission dynamics in populations where contact rates saturate as the infectious population becomes large.

The specific nonlinear incidence function (12) represents a stronger saturation effect than the Holling type II form. In this function, $\alpha$ quantifies the strength of the nonlinear inhibitory effect. Biologically, the quadratic term in the denominator implies that as the infectious population grows, the per-capita transmission rate declines more rapidly, reflecting enhanced behavioral avoidance, limited contact opportunities, or intervention measures that become increasingly effective at high infection levels. Compared with the standard Holling type II function, this formulation captures scenarios in which crowding or inhibitory effects escalate disproportionately as the epidemic progresses, providing a more realistic description of transmission dynamics in highly affected populations.

In the general nonlinear incidence function (13), the exponent *p* captures the nonlinearity of transmission with respect to the number of infectious individuals. When $p = 1$, new infections grow linearly with *I* as in the standard mass action incidence; if $p < 1$, transmission increases sublinearly, reflecting limited contact opportunities or behavioral changes as infection spreads, whereas $p > 1$ indicates superlinear growth due to clustering or cooperative effects. The exponent *q* governs the saturation or inhibitory effect on transmission when the infectious population is large: larger *q* values correspond to stronger saturation, representing crowding effects, behavioral avoidance, or intervention measures that reduce effective contacts, while smaller *q* values indicate weaker saturation. Together, *p* and *q* allow the model to capture complex infection dynamics beyond the standard mass action assumption, with *p* controlling the scaling of infection with infectives and *q* controlling how rapidly transmission saturates as infections increase.

The Beddington-DeAngelis incidence function (14) accounts for saturation effects in disease transmission arising from both susceptible and infectious populations. Here, $\alpha _1$ and $\alpha _2$ quantify the inhibitory effects due to high densities of susceptibles and infectives, respectively. Biologically, the inclusion of *S*(*t*) in the denominator reflects that a large susceptible population may limit effective contacts per individual, for example through competition for contacts or behavioral saturation, while the *I*(*t*) term in the denominator represents the usual inhibitory effect from a large infectious population, such as crowding, behavioral avoidance, or intervention measures. This functional form generalizes the mass action and the Holling type II incidence functions, providing a more realistic description of transmission dynamics in populations where both susceptible and infectious densities can influence the rate of new infections.

The interpretations of the parameters for model (9) with the incidence functions (10) to (14) are given in Table 3.

**Table 3:** Interpretations of parameters in model (9) with incidence functions (10)-(14)

Parameter	Interpretation
$\lambda$	Birth rate and death rate
$\beta (t)$	Transmission rate
*g*	Maturation rate from the non-adult stage to the adult stage
$1/\gamma$	Incubation period
$\delta$	Recovery rate
$\alpha$	Half saturation constant
*p*	Nonlinearity in infection growth with respect to infectious individuals
*q*	Degree of saturation/inhibition as infectious population increases
$\alpha _1$	saturation effect due to susceptible individuals
$\alpha _2$	saturation effect due to infectious individuals

### Extracting Measles Transmission Rates Using Inverse Method

We approximate the disease incidence $\omega (t)$ by the splined weekly new infection proportions. For the parameters in model (9), we use the same values as in Kong et al. (2015). For the parameters in the incidence functions (10) to (14), we choose a few different values based on the references (Kong et al. 2015; Capasso and Serio 1978; Xiao and Ruan 2007; Hethcote and van den Driessche 1991; Liu and Wei 2022) (see Figures 5, 6, 7, 8, 9). Then We solve the equations for each model using MATLAB ode45 and use the resulting solutions to compute the transmission rates as follows:

- For model (9) with the mass action incidence function (10):
  $$\begin{aligned} \beta (t)=\frac{\omega (t)}{S(t)I(t)}. \end{aligned}$$
- For model (9) with the Holling type II incidence function (11):
  $$\begin{aligned} \beta (t)=\frac{(1+\alpha I(t))\omega (t)}{S(t)I(t)}. \end{aligned}$$
- For model (9) with the specific nonlinear incidence function (12):
  $$\begin{aligned} \beta (t)=\frac{(1+\alpha I(t)^2)\omega (t)}{S(t)I(t)}. \end{aligned}$$
- For model (9) with the general nonlinear incidence function (13):
  $$\begin{aligned} \beta (t)=\frac{(1+\alpha I(t)^q)\omega (t)}{S(t)I(t)^p}. \end{aligned}$$
- For model (9) with the Beddington-DeAngelis incidence function (14):
  $$\begin{aligned} \beta (t)=\frac{(1+\alpha _1S(t)+\alpha _2I(t))\omega (t)}{S(t)I(t)}. \end{aligned}$$

### Comparing Measles Transmission Rates

**Fig. 3:**
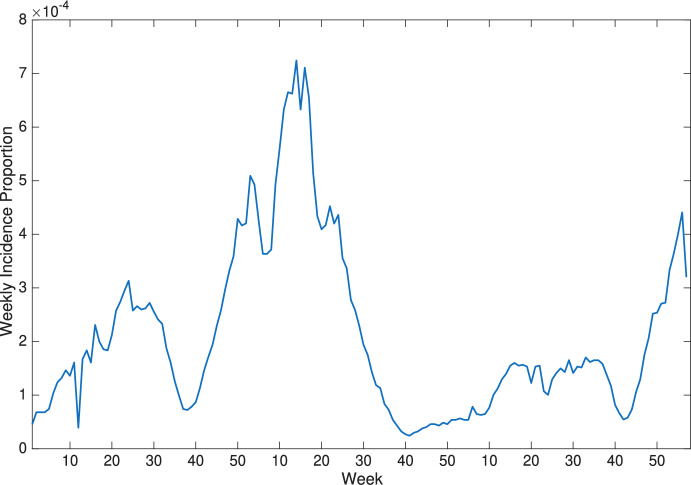
Weekly reported measles incidence proportion in England and Wales from 1950 to 1952. Data source: Bolker (2025)

In this section, we use the results from Kong et al. (2015) as a baseline for comparison with the transmission rates obtained from our model with different incidence functions. Following (Kong et al. 2015), we set the initial values as $S(0)=0.2$, $E(0)=0.003$, $I(0)=0.003$, $A(0)=0.79$ and take $\delta =0.0003$, $a=1$, $v=1$, $g=0.0012$, $N=4.4\times 10^7$, where *N* is used to obtain the incidence proportion from incidence data (i.e., incidence proportion = incidence data/*N*). The weekly reported measles incidence proportion shown in Figure 3 is used as the observational data to estimate measles transmission rates.

**Fig. 4:**
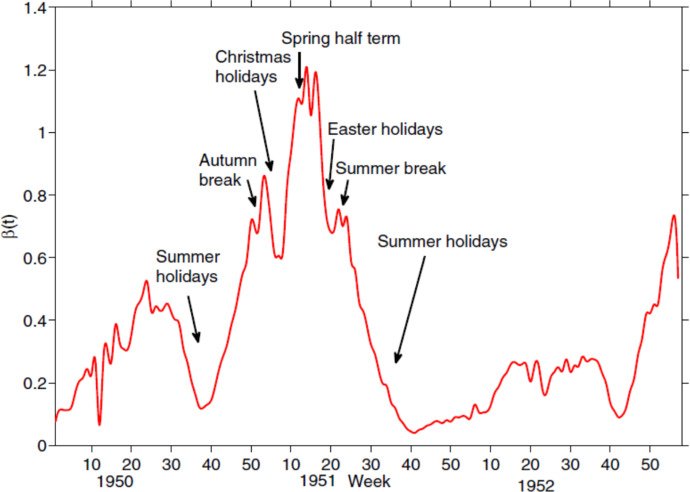
England and Wales time-dependent measles transmission rate from 1950-1952 estimated for model (9) with the mass action incidence (10). Adopted from Kong et al. (2015)

Figure 4, adopted from Kong et al. (2015), shows the time-varying transmission rate $\beta (t)$ for measles obtained using the continuous inverse method. This figure demonstrates the reasonableness of the estimated transmission rates, as the peaks and troughs align well with key dates and school holidays, reflecting realistic temporal variations in measles transmission. Specifically, peaks in $\beta (t)$ correspond to school terms, when contact rates among children are high, while sharp declines occur during periods such as the Christmas, Easter, and summer holidays, when school closures reduce contact frequency.

**Fig. 5:**
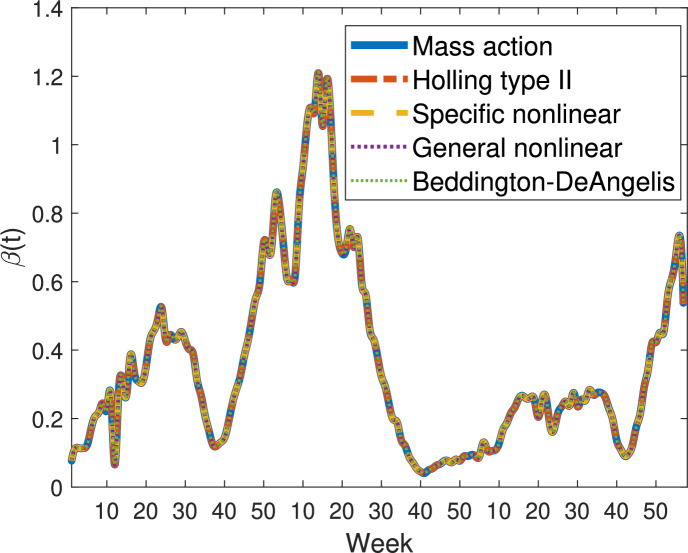
Comparison of the estimated transmission rates for model (9) with different incidence functions (10) to (14), where $\alpha =0.005$, $p=1$, $q=0.5$, $\alpha _1=0.005$ and $\alpha _2=0.5$. Blue solid curve: $\beta (t)$ with the mass action incidence (10); Orange dash-dotted curve: $\beta (t)$ with Holling type II incidence (11); Yellow dashed curve: $\beta (t)$ with the specific nonlinear incidence (12); Purple dotted curve: $\beta (t)$ with the general nonlinear incidence (13); Green dotted curve: $\beta (t)$ with Beddington-DeAngelis incidence (14)

In Figure 5, we present the transmission rates for model (9) with incidence functions (10) to (14), with parameters set as $\alpha =0.005$, $p=1$, $q=0.5$, $\alpha _1=0.005$ and $\alpha _2=0.5$. Remarkably, the transmission rate curves for all incidence functions overlap almost completely, indicating that, under these parameter values, the model’s inferred transmission rates are robust to the specific functional form of the incidence term.

**Fig. 6:**
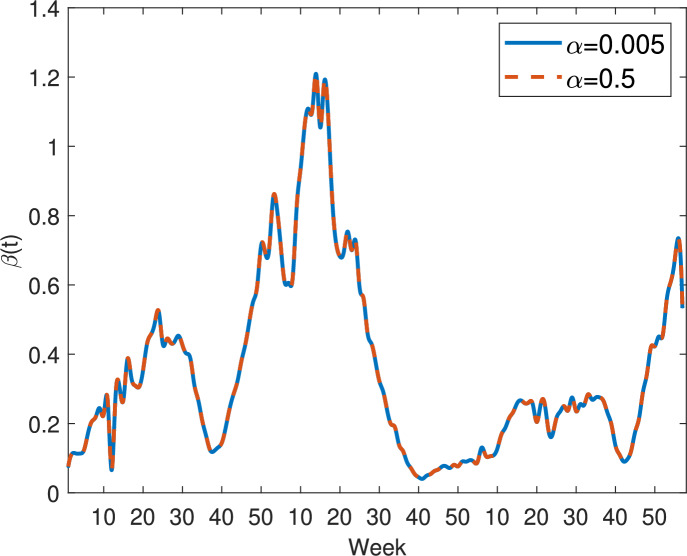
Transmission rates estimated from model (9) with Holling type II incidence (11). Blue solid curve: $\alpha =0.005$; Orange dashed curve: $\alpha =0.5$

**Fig. 7:**
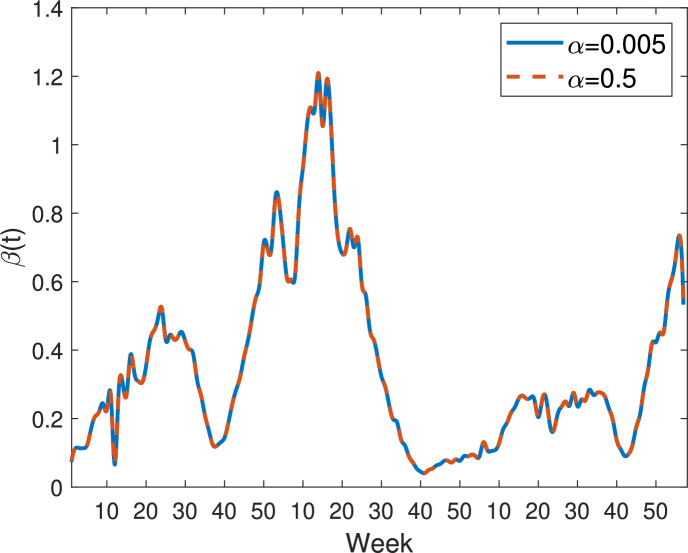
Transmission rates estimated from model (9) with the specific nonlinear incidence (12). Blue solid curve: $\alpha =0.005$; Orange dashed curve: $\alpha =0.5$

Figures 6 and 7 compare the transmission rates estimated from model (9) with two different nonlinear incidence functions: Holling type II incidence (11) in Figure 6 and the specific nonlinear incidence (12) in Figure 7, with half-saturation parameter values $\alpha = 0.005$ (blue solid curves) and $\alpha = 0.5$ (orange dashed curves). In both cases, the curves nearly overlap, indicating that the estimated transmission rates are largely insensitive to the value of $\alpha$ within this range. This suggests that, for these incidence formulations, variations in the saturation effect have minimal impact on the inferred temporal dynamics, and the overall transmission patterns are primarily determined by the underlying contact structure and disease progression rather than the specific parameter choice.

**Fig. 8:**
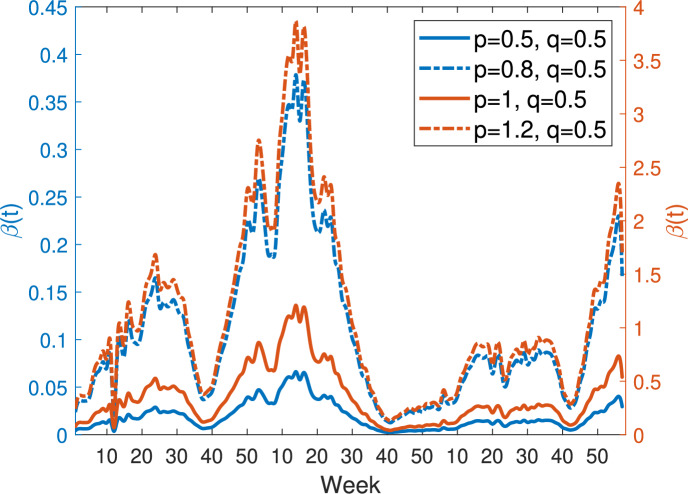
Transmission rates estimated from model (9) with the general nonlinear incidence (13) when $q=0.5$, $\alpha =0.005$. Blue solid curve: $p=0.5$; Blue dash-dotted curve: $p=0.8$; Orange solid curve: $p=1$; Orange dash-dotted curve: $p=1.2$

Figure 8 shows the transmission rates estimated from model (9) with the general nonlinear incidence function (13) and $q = 0.5$, $\alpha = 0.005$, for different values of the parameter *p*: $p = 0.5$ (blue solid curve), $p = 0.8$ (blue dash-dotted curve), $p = 1$ (orange solid curve), and $p = 1.2$ (orange dash-dotted curve). As *p* increases, the overall magnitude of the estimated transmission rates also increases. However, all curves exhibit the same monotonic pattern, with peaks and troughs occurring at the same times, indicating that variations in *p* primarily scale the transmission intensity without altering the timing of temporal fluctuations.

**Fig. 9:**
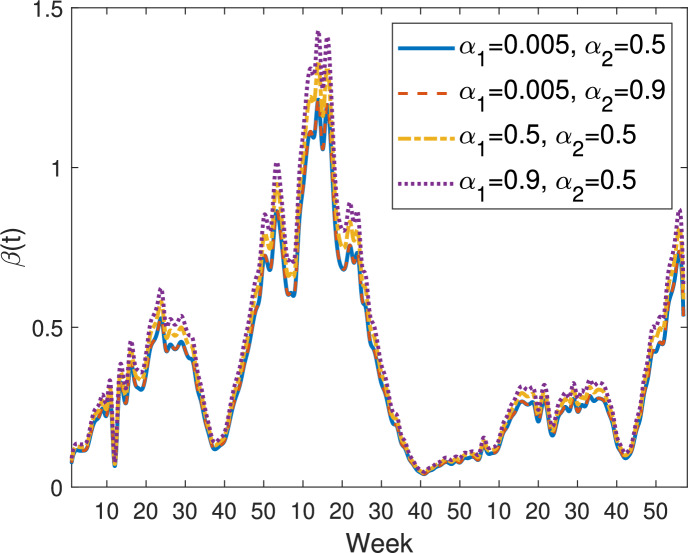
Transmission rates estimated from model (9) with the Beddington-DeAngelis incidence (14). Blue solid curve: $\alpha _1=0.005$, $\alpha _2=0.5$; Orange dashed curve: $\alpha _1=0.005$, $\alpha _2=0.9$; Yellow dash-dotted curve: $\alpha _1=0.5$, $\alpha _2=0.5$; Purple dotted curve: $\alpha _1=0.9$, $\alpha _2=0.5$

Figure 9 shows the transmission rates estimated from model (9) with Beddington-DeAngelis incidence function (14), for different values of the saturation parameters $\alpha _1$ and $\alpha _2$: $\alpha _1 = 0.005$, $\alpha _2 = 0.5$ (blue solid curve), $\alpha _1 = 0.005$, $\alpha _2 = 0.9$ (orange dashed curve), $\alpha _1 = 0.5$, $\alpha _2 = 0.5$ (yellow dash-dotted curve), and $\alpha _1 = 0.9$, $\alpha _2 = 0.5$ (purple dotted curve). When $\alpha _1$ is fixed and $\alpha _2$ varies, the blue solid and orange dashed curves almost completely coincide, indicating that changes in the saturation effect due to infectious individuals have minimal impact on the estimated transmission rates. When $\alpha _2$ is fixed and $\alpha _1$ varies (see the yellow dash-dotted and purple dotted curves), a larger $\alpha _1$ leads to a slightly higher transmission rate $\beta (t)$, but the curves share the same monotonicity, with peaks and troughs occurring at the same times. This indicates that the saturation effect due to susceptible individuals primarily influences the magnitude of transmission, while the timing of temporal fluctuations is robust and mainly governed by other factors.

An investigation of the condition under which the transmission rates have the same monotonicity is provided in Appendix 1.

## Discussion

In this study, we compared time-varying transmission rates $\beta (t)$ extracted from epidemic data using the continuous inverse method across models with different compartmental structures and incidence functions. To assess the impact of model structure, we compared the transmission rates of SIS, SIR, SEIS, SEIR, SISV, SIRV, SEISV, and SEIRV flu models. To examine the effect of incidence function, we analyzed measles models with the same model structure but different incidence formulations, including mass action, Holling type II, a specific nonlinear, a general nonlinear, and Beddington-DeAngelis forms. These models were selected because they capture the essential dynamics of epidemic spread and include both classic and more mechanistically grounded formulations, making them broadly relevant to a wide range of infectious diseases. Despite these structural and functional variations, the estimated transmission rates exhibited remarkably similar monotonic patterns, with consistent timing of peaks and troughs. This consistency demonstrates the robustness of the inverse method in capturing temporal transmission dynamics across different model assumptions, indicating that the inferred transmission rates are primarily determined by the information contained in the observed infection data. Importantly, the patterns observed in the extracted transmission rates, such as the rise-and-fall ordering across incidence functions, are expected to generalize to other compartmental structures and incidence forms not explicitly analyzed here.

Given the demonstrated robustness of the inverse method, we can be confident in using it to estimate transmission rates from observed incidence data. These estimates provide a reliable quantitative bridge between compartmental differential equation models and machine learning approaches, thereby enabling more accurate forecasting of infectious disease dynamics (see, e.g., Wang and Wang (2024); Wang et al. (2022a)) and highlighting the practical value of the inverse method in integrated modeling frameworks.

Nevertheless, some limitations remain. Our analysis was confined to deterministic ordinary differential equation models without explicit representation of stochastic fluctuations or spatial effects. Furthermore, a key assumption of our approach is that reported cases represent the “ground-truth” incidence of infection. However, as noted in previous studies, reported case data are subject to at least two important limitations: imperfect observation and right truncation (Lipsitch et al. 2015; Gostic et al. 2020; De Salazar et al. 2022). Imperfect observation arises because not all infections are detected or reported, leading to underestimation of true incidence. Right truncation occurs because cases occurring near the end of the observation period may not yet have been reported, which can bias estimates of time-varying transmission rates. These limitations may reduce the accuracy and interpretability of inferred transmission parameters.

Future work could evaluate the robustness of the methods under scenarios of incomplete reporting and delayed case reporting, which would help better characterize the uncertainty associated with using reported cases as a proxy for true incidence. Moreover, the present framework could be extended to stochastic and spatial models, and applied to empirical datasets of other diseases to further assess and validate the robustness of the approach observed here.

## Data Availability

The data used in this study are publicly available from the sources cited in the manuscript.

## Appendix 1

In this Appendix, we investigate the condition under which the transmission rates of model (9) with incidence functions (10) and (11) exhibit the same monotonicity. The conditions for the transmission rates of model (9) with other incidence functions to share the same monotonicity can be derived using a similar approach.

### Theorem 1

Let $f_1(t)=\frac{\omega (t)}{S(t)I(t)}$ and $f_2(t)=\frac{(1+\alpha I(t))\omega (t)}{S(t)I(t)}$. If $|\alpha f_1 I'| \le (1+\alpha I)\,|f_1'|$, then the transmission rates for model (9) with the mass action incidence function (10) and the Holling type II incidence function (11) have the same monotonicity.

### Proof

Since

$$\begin{aligned} f_1(t)=\frac{\omega (t)}{S(t)I(t)}, \ \ f_2(t)=\frac{(1+\alpha I(t))\omega (t)}{S(t)I(t)}, \end{aligned}$$

we have

$$ f_2(t) = \bigl (1+\alpha I(t)\bigr )f_1(t) := h(t)\,f_1(t), \qquad \text {where}\ h(t)=1+\alpha I(t)>0, $$

so

$$ f_2' = h\,f_1' + \alpha f_1 I':=ha+b, \quad \text {where}\ a=f_1',\ b=\alpha f_1 I'. $$

To ensure $\operatorname {sign}(f_1')=\operatorname {sign}(f_2')$, i.e., $\operatorname {sign}(a)=\operatorname {sign}(h a + b)$, it suffices to bound the “perturbation” $b$ so that it cannot flip the sign of *ha*. A sufficient condition is

$$ |b| \le h\,|a| \quad (\text {strict inequality gives strict monotonicity}). $$

Indeed, if $a>0$, then

$$ h a + b \ge h a - |b| \ge 0. $$

If $$a<0$$, then

$$ h a + b \le h a + |b| \le 0. $$

Thus, the sign of $f_2'$ matches that of $f_1'$.

Substituting back $a=f_1'$, $b=\alpha f_1 I'$ and $h=1+\alpha I$, we obtain a sufficient condition for $f_1$ and $f_2$ to have the same monotonicity as follows:

$$\begin{aligned} |\alpha f_1 I'| \le (1+\alpha I)|f_1'|. \end{aligned}$$

$$\square $$

If $f_1\ne 0$, we can divide the above inequality by $|f_1|$ to obtain

$$ |\alpha I'| \le \bigl (1+\alpha I\bigr )\,\Bigl |\frac{f_1'}{f_1}\Bigr |. $$

On the left side, $|\alpha I(t)'|$ measures how fast the infectious population *I*(*t*) is changing, scaled by the saturation parameter $\alpha$. This represents the speed of epidemic growth or decline when it has the nonlinear incidence function. On the right side, $(1+\alpha I(t))\cdot \bigl |f_1'(t)/f_1(t)\bigr |$ is essentially the relative growth rate of the baseline transmission rate $f_1(t)$, weighted by the saturation term. This captures how quickly the transmission environment itself is changing (e.g., due to climate, behavior, or policy). This implies that the epidemic curve *I*(*t*) (the number of infectious individuals) must not change too fast compared to the relative change in the underlying transmission environment (e.g., contact rate, seasonality, external drivers). Otherwise, the nonlinear correction in $f_2$ (from the $(1+\alpha I)$ saturation) can flip the monotonicity relative to $f_1$.
